# A systematic review of informal supporters of intimate partner violence survivors: the intimate partner violence model of informal supporter readiness

**DOI:** 10.7717/peerj.15160

**Published:** 2023-05-09

**Authors:** Ryan L. Davies, Kylie Rice, Adam J. Rock

**Affiliations:** University of New England, Arimidale, New South Wales, Australia

**Keywords:** Intimate partner violence, Domestic violence, Informal supporter, Social network, Help-giving, Intimate partner violence theory

## Abstract

**Background:**

Intimate partner violence (IPV) is a serious public health issue that consists of physical, sexual, and psychological violence perpetrated by a current or former partner. Informal supporters (*e.g.*, family and friends) of survivors are more often witness to IPV or are the first people a survivor will disclose abuse to and are more able to provide consistent ongoing support than professional services. Therefore, greater understanding of informal supporters is warranted to aid in reducing the risks experienced by survivors. This systematic review aimed to: (1) identify factors associated with either an increase or decrease in helping behaviour toward a survivor, (2), identify the most effective self-care strategies employed by informal supporters, and (3) consider the current theoretical approaches used to understand informal supporters help-giving behavioural intention.

**Methods:**

A systematic literature search was conducted following the PRISMA guidelines. The search included English language articles published between 2005 and 2021 in the databases Psych Articles, Scopus, Proquest Social Services Abstracts, and Ebscohost. Studies were included if the primary research aims explored the motivators and inhibitors of helping intention or self-care strategies of adult social network members of adult IPV survivors. Two reviewers independently screened all identified articles for inclusion suitability.

**Results:**

One hundred and twenty articles were subjected to full text screening resulting in 31 articles being identified as meeting inclusion criteria. Synthesis of the findings identified the following three key areas associated with help-giving behavioural intentions: normative factors, individual factors, and situational factors. There were no articles identified that considered self-care of informal supporters. Of the 31 articles, 22 had a theoretical underpinning. None of the utilised theories explained all three of the identified factors of help-giving behavioural intention.

**Conclusion:**

These results are integrated into a proposed Intimate Partner Violence Model of Informal Supporter Readiness (IPV-MISR), incorporating the identified factors associated with help-giving behavioural intention. This model provides a framework for conceptualising the readiness of an informal supporter to provide adequate support to IPV survivors. The model extends existing theoretical standpoints and has utility in both practice and research.

## Introduction

Intimate partner violence (IPV) is a serious issue confronted by people of all social groups around the world. Whilst men can be victims of IPV and women perpetrate violence, women are four times more likely to experience IPV than men (Australian Bureau of Statistics, 2016). It is estimated that about one in four women over the age of 15 years have experienced violence from a current or former intimate partner as either physical, sexual, or psychological violence (including coercive control) or a combination of these (Devries et al., 2013). The lifetime prevalence rates of IPV against women range from 20% in the Western Pacific region, 22% in Europe, 25% in the Americas, to 33% in Africa and South-East Asia (World Health Organisation, 2021). Consequently, IPV accounts for over half of all female homicides (United Nations Office of Drugs and Crime, 2020). Help-seeking behaviours, including barriers, of survivors have been widely considered (for a review see Lelaurain, Graziani & Lo Monaco (2017)) and have identified social support as being routinely utilised by survivors for support. The importance of informal supporters was highlighted in a review by Sylaska & Edwards (2013); however, there has not been a systematic review considering the perspectives of informal supporters themselves.

### Intervention and support from social networks

Survivors of IPV have two main streams of support that can help reduce the risks and harm associated with IPV—formal and informal supporters (Edwards & Dardis, 2016). Formal supporters are professionals such as police, domestic violence outreach services, health professionals, and counsellors. In contrast, informal supporters are members of the survivor’s social network, and include family members, friends, neighbours, and colleagues (Sylaska & Edwards, 2013). The two support streams can be utilised independently or in a complementary manner as needed by the survivor.

The types of support that can be offered to survivors which aim to reduce the risk of harm from IPV are typically conceptualised by public health models in three levels of intervention—primary, secondary, and tertiary (Coker et al., 2004; García-Moreno et al., 2015; Kirk et al., 2017). Primary interventions aim to prevent the onset of violence, secondary interventions aim to intervene and interrupt existing violence, and tertiary interventions aim to prevent the harm and risks associated with violence that has already occurred (García-Moreno et al., 2015). In the context of informal support, the majority of interventions occur as either secondary or tertiary responses and as such are the focus of this systematic review.

Secondary interventions relate to the actions taken when directly witnessing acts of IPV. Commonly conceptualised as ‘bystander behaviours’ and born from bystander theory as proposed by Darley & Latané (1968) these occur generally in three distinct streams of (1) physically or verbally intervening to protect the victim, (2) seeking additional assistance such as calling the police, or (3) ignoring the behaviour (Gracia et al., 2018). Given the private nature of IPV, secondary interventions are not commonly provided by formal supports who have limited opportunity to directly witness acts of IPV. Informal supporters, however, are generally more exposed to the occurrence of IPV behaviours and as such have a greater opportunity to provide secondary interventions (Taylor et al., 2016). However, bystander theory itself does not account for some of the contextual factors present in IPV, such as the influence of the bystander relationship with either the survivor or perpetrator.

Further, while there are numerous avenues for formal tertiary support post incidents of IPV (*e.g.*, police, domestic violence outreach services, health professionals, counsellors), many survivors are reluctant to make a disclosure to professionals. There are many challenges survivors face when making the decision to disclose their abuse to a professional support, including the survivor’s perceptions of safety, concerns for confidentiality (Heron & Eisma, 2021), and fear of being negatively judged (Ansara & Hindin, 2010). Therefore, an estimated 75% of IPV survivors make initial disclosures to informal networks, such as family members, friends, colleagues, and neighbours (Ansara & Hindin, 2010).

Once an initial disclosure has been made, informal supporters are able to assist survivors with emotional support (*e.g.*, listening to the survivor and validating their emotional experience), informational (*e.g.*, providing useful advice and suggestions to cope), or instrumental support (*e.g.*, providing tangible aid such as money or refuge) (Mahapatro, Prasad & Singh, 2021; Sylaska & Edwards, 2013). These support behaviours can subsequently reduce the barriers and risks associated with accessing formal supports (Davies, Rice & Rock, 2022; Evans & Feder, 2015). Unfortunately, responses from informal supporters to disclosures are not always helpful to the survivor. Negative responses to disclosure can include victim blaming, minimizing, and refusing to believe the survivor. When interviewing victims of IPV, Trotter & Allen (2009) found that it was not uncommon for informal supporters to respond with disbelief, shock, or to side with the perpetrator, which had negative consequences for the survivor. Further, it is noted that barriers such as substance use, childcare, and mental health can inhibit the ability of an informal supporter (Latta & Goodman, 2011).

Unplanned disclosures (*i.e.,* those made after direct questioning) are also common among informal supporters and are comparatively rare within formal support settings (Fisher et al., 2020). Informal supporters have ongoing contact and familiarity with survivors that formal supporters do not and, therefore, may be more perceptive to indicators of abuse. The closeness of the relationship between the informal supporter and the survivor results in greater direct questioning about IPV and, in turn, unplanned disclosures (Sylaska & Edwards, 2013).

### Benefits of informal support networks

The importance of social supports as a factor in reducing the risks associated with IPV have long been recognised (Carlson et al., 2002). Informal support networks have been shown to have positive implications for survivors even in the absence of formal support systems. First, strong social supports are a key protective factor in reducing the likelihood of a women experiencing violence in her relationship (Capaldi et al., 2012). Second, should a women experience an episode of violence, higher levels of social support are associated with reduced risk of future victimisation (Goodman et al., 2005). In a longitudinal study on re-abuse rates, Goodman et al. (2005) found women with higher levels of social support experienced a 20% risk of re-abuse in a 12-month period compared to a 60% risk of re-abuse for women with lower levels of social support. It has also been found that disclosure to formal supports was associated with greater risk of a survivor experiencing subsequent injury from violence compared to disclosing to informal supporters (Belknap et al., 2009). Additionally, women who have left an abusive relationship are significantly less likely to experience abuse in future intimate relationships if they have a strong social support network (Plazaola-Castano, Ruiz-Perez & Montero-Pinar, 2008).

Furthermore, informal supporters offer continued benefits to survivors who experience ongoing IPV. Broadly, IPV survivors who perceived having strong social supports reported increased quality of life (QoL) outcomes in areas such as physical health, vocational attainment and achievement, and sense of belonging, compared to women who reported weaker social networks (Beeble et al., 2009). QoL outcomes appear to be strengthened by support networks facilitating women to utilise appropriate coping strategies and skills. Positive coping strategies are also associated with better reported mental health outcomes (Coker et al., 2004). Additionally, through being supported, women have reported less self-blame and negative affect (Latta, 2009). For example, in a cross-sectional survey of 621 women experiencing IPV, higher levels of social support were associated with reduced risk of depression, anxiety, post-traumatic stress disorder, and suicidal ideation (Coker et al., 2004).

### The wellbeing of informal supporters

Although a primary modality of support for an IPV survivor, being an informal supporter comes with challenges. The role of an informal supporter can be taxing given that the need to provide support is unpredictable. In a qualitative study of 23 participants in the UK, Gregory et al. (2017) identified a number of acute and chronic psychological and physical symptoms. These symptoms included increases in anxiety, anger, depression, sleep difficulties, and appetite loss. In a first of its kind study, Sigurvinsdottir, Riger & Ullman (2016) identified that emotional distress uniquely predicated increased symptoms of PTSD in informal supporters. Given the prevalence of IPV, and therefore the number of people who are providing informal support, further understanding of informal supporter self-care is warranted.

### Objectives

As the field of IPV research has evolved, a deeper understanding of how survivor’s engage informal supporters to create safety and the benefits of strong social networks in reducing ongoing risks of IPV has been developed. However, research with a primary focus on perspectives of informal supporters is a relatively new area of research. Consequently, there is currently no conceptual model of the factors which influence an informal supporter’s decision to support IPV survivors. As such, this systematic review aimed to consider the gaps in understanding of informal supporters of IPV survivors, and consisted of the following objectives:

(a) To identify factors associated with either an increase or decrease in the likelihood of an informal supporter helping a survivor of IPV
(b) To identify informal supporter self-care strategies associated with greater psychological wellbeing
(c) To identify the existing theories utilised to understand an individual’s likelihood of helping a survivor of IPV

## Materials & Methods

This systematic review was developed in accordance with the Preferred Reporting Items for Systematic Reviews and Meta-analyses (PRISMA; Page et al., 2021) guidelines. The protocol that informed the systematic review was registered with the International Prospective Register of Systematic Reviews (PROSPERO, 2021; CRD42021288834). A systematic review, rather than a meta-analysis, was performed as the identified studies were heterogeneous in terms of (i) study design, (ii) methodology, and (iii) the variables measured. Given the issues of heterogeneity, the main findings identified in each study were consolidated through a narrative synthesis (Siddaway, Wood & Hedges, 2019), using the Synthesis Without Meta-analysis (SWiM) reporting guidelines (Campbell et al., 2020). The findings of the narrative synthesis were organised into thematic groups. The methods used to define eligibility and screening are outlined below.

### Eligibility criteria

Studies that met the following criteria were included: (1) the study had first-hand consideration of social network members (*e.g.*, friends, family members, colleagues) and not indirect reports from IPV survivors, (2) the study investigated the motivators and/or inhibitors of intention to help with either secondary interventions (*i.e.,* bystander actions in response to directly witnessing an IPV act) or tertiary interventions (*i.e.,* support following a disclosure of IPV), (3) both the informal supporter and the IPV survivor were adults (older than 16 years), (4) the IPV survivor identified as female, given the vast majority of this type of violence is perpetrated against women, (5) the study was published in English or an English translation was available. There were no exclusion criteria related to study type or geographic population. IPV was defined as physical, sexual, and/or psychological abuse (including coercive control) by a current or former partner (Jewkes, 2002).

### Information sources

During November and December 2021, searches were conducted using four databases: PsychARTICLES, Scopus, ProQuest Social Services Abstracts, and EbscoHost, for articles published between 2005 and 2021. Only studies from 2005 onwards were included due to developments in the field at that time that included the perspective of the informal supporter in addition to that of the survivor. Identified articles were imported to Endnote and then saved to the Covidence systematic review software (Veritas Health Innovation, 2021). Table 1 presents a summary and overview of the included studies.

**Table 1:** Summary of studies selected.

Study	Country	Sample	Design	Theory	Intervention level	Intended or actual behaviour	Focus of study	Findings
Abramsky et al. (2018)	Uganda	2,532 (45% female)	Experimental	Bystander theory	Secondary and tertiary interventions	Actual behaviour	To explore the role of community members in IPV prevention and response, both within and outside the context of a holistic community intervention.	Results show that older age, longer length of own relationship, longer time living in community, and attitudes that condemn IPV were associated with increased willingness to help.
Amar, Sutherland & Kesler (2012)	USA	202 college students (70% female; age range 18–22 years)	Experimental	Bystander theory	Secondary and tertiary interventions	Behavioural intention	This study evaluated the effectiveness and feasibility of a bystander education program that was adapted to a specific university setting.	The program increased participants confidence in how to respond to IPV situations, lowered rape myth acceptance, increased awareness of IPV as a problem, increased sense of responsibility, and increased willingness to intervene in IPV situations.
Amar, Sutherland & Laughon (2014)	USA	157 college students (53% female; age range 18–24 years)	Correlational	Bystander theory	Secondary and tertiary interventions	Behavioural intention and actual behaviour	This study compared male and female college students on rape attitudes, bystander efficacy, intention to act as a bystander, barriers to acting as a bystander, and actual use of bystander behaviours.	Gender was a significant factor for reducing rape attitudes, increasing bystander confidence, and bystander behaviours. Men had more negative rape attitudes and less bystander confidence and reported lower intention to engage in bystander behaviours. Participants were more hesitant to respond when either the survivor or perpetrator was an unknown person.
Baldry, Pacilli & Pagliaro (2015)	Italy	303 university students (50.5% female; mean age = 26.49 years, *SD*= 7.69)	Correlational	Moral disengagement theory	Secondary and tertiary intervention	Behavioural intention	This study focused on bystander’s reactions towards a victim of an IPV episode to try to discover whether and how a specific form of prejudice (infra-humanization) might hold toward the victim and may lead to moral disengagement resulting in decreased willingness to help the female survivor.	Participants were less willing to report an IPV episode and support the survivor when she had admitted an affair than when she had not admitted an affair. Participants attributed fewer secondary emotions to the victim when she had admitted an affair than when she had not admitted an affair. Therefore, admitting an affair with another man determined the infra-humanization of the victim. The effect of condition (admitting / not admitting; IV) on the willingness to report the IPV episode and support the victim (DV) was reduced when perceived humanness (Mediator) was entered into the equation.
Baldry & Pagliaro (2014)	Italy	218 college students (79% female; mean age = 22.5 years, *SD*= 4.48)	Experiment	Social identity theory	Secondary and tertiary interventions	Behavioural intention	The aim of this study was to explore what role exposure to a helping-norm (*i.e.*, it is morally correct to intervene, call the police, help) or a not-helping-norm (*i.e.*, belief that IPV is a private matter, not to be interfered with) has on intention to help.	When confronted with a norm that suggests that helping a victim of IPV is morally right, respondents in turn increased their reported willingness to help the victim when they strongly identified with the in-group. Importantly, evaluations of the episode of IPV did not alter the pattern of results, thus confirming that willingness to help a survivor is influenced by shared social norms.
Banyard (2008)	USA	389 college students (70% female; mean age = 19.3 years, *SD*= 1.2)	Correlational	Bystander theory	Secondary and tertiary interventions	Behavioural intention and actual behaviour	This study was exploratory and focussed on the development of measures of bystander attitudes and behaviour in the context of IPV. It also examined correlates of these bystander behaviours.	Results showed that being female, knowing a survivor of sexual violence, higher levels of extroversion, interpersonal and socio-political control, greater perceived sense of community, greater knowledge of information about sexual violence, and lesser rape myth acceptance were associated with more positive bystander outcomes.
Banyard & Moynihan (2011)	USA	406 college students (51.4% female; mean age = 18.7 years, *SD*= 1.29)	Correlational	Bystander theory	Secondary and tertiary interventions	Actual behaviour	This study aimed to increase the understanding of variability in bystander behaviour among student populations who are likely targets of prevention programs, applying the bystander framework.	Results showed that being younger, a greater sense of responsibility for ending violence, greater perceived efficacy to be an effective bystander, and having a score on the decision balance scale with pros outweighing cons, was correlated with a higher overall number of IPV related bystander intervention behaviours.
Beeble et al. (2008)	USA	6,010 adults (55% female; Mean age = 42.33 years, *SD*= 14.93)	Correlational	N/A	Secondary and tertiary interventions	Actual behaviour	This study aimed to identify factors that relate to individuals’ willingness to help survivors of IPV.	Results show that being female, younger, having a greater perception that IPV is a problem in the community, viewing IPV as a criminal justice issue, having prior experience of violence (*i.e.*, witnessing violence as a child and experiencing IPV as an adult) were associated with greater willingness to intervene.
Bovill & White (2020)	UK	1,604 college students (65.5% female; age range 18–21 years).	Experimental	Bystander theory	Secondary and tertiary interventions	Actual behaviour	This study explored the relationship between awareness of IPV, confidence to intervene, and self-efficacy on positive action. Additionally, it examined if participation in a bystander intervention was associated with raised levels of awareness.	Results show that both awareness and confidence are individually and jointly significantly related to the number of positive actions, and confidence in dealing with IPV partially mediates the relationship between awareness and the number of positive actions.
Cascardi et al. (2018);	USA	556 college students (77.4% female; mean age = 20.15 years, *SD*= 2.98)	Correlational	Bystander theory	Secondary and tertiary interventions	Actual behaviour	The primary aims of this study were to (a) use CFA to evaluate the factor structure of the Bystander Behaviour Scale (BBS) in a sample of university undergraduates and (b) test associations between prior victimization (general and family-specific) and BBS factors.	The (1) *Proactive Behaviours* factor was positively associated with both general and family-specific prior victimization. The (2) *Risky Situations* and (3) *Party Safety* factors were positively associated with general prior victimization but were not associated with family-specific prior victimization. The (4) *Accessing Resources* factor was not associated with either general or family-specific prior victimization.
Chabot et al. (2009)	USA	71 college students (63% female; age range 18–28 years).	Correlation	Bystander theory	Secondary and tertiary interventions	Behavioural intention	This study assessed the role of situational and personal variable to understand intervention decision of informal helpers in IPV situations.	Results show that experience of child abuse, male gender of perpetrator, higher severity of abuse, and attributions of perpetrators actions to drunkenness were associated with willingness to intervene.
Cinquegrana, Baldry & Pagliaro (2018)	Italy	464 adults (66.5% female; mean age = 35.83 years; *SD*= 13.99)	Experimental	Attribution theory	Secondary intervention	Behavioural intention	This paper aimed to investigate the influence of contextual factors on the attribution of responsibility to female survivors of an IPV episode.	Participants attributed more responsibility to the survivor in the infidelity condition and were less willing to intervene. Participants with traditional, misogynistic, and sexually hostile gender role attitudes attributed greater responsibility to the survivor in the infidelity condition and were less willing to intervene.
Edwards & Dardis (2016)	USA	743 college students (69% female; age range 18–29 years)	Correlational	Attribution theory	Tertiary intervention	Actual behaviour	Guided by the attribution framework, this study assessed factors (i.e., situation-specific, individual, relational, attributional, and emotional response) related to positive and negative reactions from the perspective of disclosure recipients.	The following factors were associated with positive social reactions: (a) *situation-specific factors*: the survivor being a woman and greater frequency of IPV incidents disclosed by the victim, (b) *individual factors*: greater frequency of lifetime IPV incidents reported by the disclosure recipient, and less accepting attitudes toward IPV, (c) *relational factors*: a closer relationship with the victim and less close relationship with the perpetrator, (d) *attributional*: attributing less survivor responsibility, (e) *emotional response factors*: greater survivor empathy, and greater emotional distress experienced by the disclosure recipient at the time of disclosure.
Edwards et al. (2014)	USA	203 adults (67.4% female; mean age = 21.05 years, *SD*= 1.93)	Correlational	Social disorganisation theory	Secondary and tertiary interventions	Actual behaviour	This study examined the extent to which community-level poverty rates and collective efficacy was associated with individual reports of IPV perpetration, victimization, and bystander intervention.	Results showed that collective efficacy (helping norm) was positively related with bystander intervention while individual-level income status was negatively related. Community-level poverty was unrelated.
Fenton & Mott (2018)	United Kingdom	354 college students	Experimental	Bystander theory	Secondary and tertiary interventions	Behavioural intention and actual behaviour	This study aimed to provide an evaluation of the effectiveness in a student-led awareness raising campaign.	Results showed significant improvement in the desired direction in rape myth acceptance; IPV myth acceptance; bystander efficacy; readiness to help (both denial and responsibility). They showed significant improvement in the desired direction in the measure for intent to help.
Franklin, Brady & Jurek (2017)	USA	377 college students	Correlational	N/A	Secondary intervention	Behavioural intention	Aimed to explore the impact of intrapersonal characteristics, and rape- and bystander-related attitudes on direct intervention in IPV situations.	Positive bystander attitudes and violence prevention efficacy was associated with increased intention to intervene for SA. While, positive bystander attitudes, personality extroversion, and exposure to a victim were associated with increased intention to intervene for IPV.
Frye (2007)	USA	199 adults (42% female)	Correlational	N/A	Secondary intervention	Behavioural intention	This study examined factors related to predicted informal social control of IPV (*i.e.*, intervening in such situations at the individual level).	Personal attitudes that are less tolerant of IPV, self-efficacy to respond to IPV, and being married were significantly positively associated with enacting informal social control. Whereas legal cynicism was significantly negatively associated. Additionally, none of the perceived social cohesion, visible disorder, social ties, organizational involvement, or social support factors tested were associated with predicted informal social control of IPV.
Gainsbury, Fenton & Jones (2020)	United Kingdom	83 adults (74% female; 16–73 years)	Experimental	Bystander theory	Secondary and tertiary interventions	Behavioural intention and actual behaviour	The aim was to evaluate the acceptability and potential utility of the first UK DVA bystander intervention within general communities.	There was a statistically significant change in the desired direction across Myth Acceptance (self and perception of peers), Bystander Efficacy, Behavioural Intent (self and perception of peers) and Perceived Law Knowledge at post.
La Ferle, Muralidharan & Kim (2019)	India	120 adults (42.5% female; mean age 43.77 years)	Experimental	N/A	Secondary intervention	Behavioural intention	The study explored the effectiveness of negative emotions (i.e., guilt and shame) on attitude toward an IPV ad and reporting intention of bystanders in India.	Overall, guilt and shame ads were more impactful on reporting intention than the control. Shame was more effective for individuals with an interdependent self-view and that individuals with an independent self-view were indifferent to the presence or absence of negative emotions in ads.
Latta & Goodman (2011)	USA	18 adults (89% female; age range 23–60 years)	Qualitative	Grounded theory	Secondary and tertiary interventions	Actual behaviour	This study uses grounded theory to explore in-depth network members’ subjective experience of learning about and responding to loved ones involved in IPV.	Three stages were identified as follows: 1. Becoming Aware**Survivor conditions:** level of violence in relationship**Mutual Conditions:** helper/Survivor relationship (physical proximity; emotional closeness), level of readiness 2. Developing a narrative**Survivor conditions:** Relationship (level of violence; children; substance use; infidelity), Personal Factors (History of mental illness; History of abuse)**Helper Conditions:** Belief about IPV, History of abuse **Mutual Conditions:** helper/survivor relationship **Others’ Reactions** 3. Taking Action**Helper Conditions:** Risk level, personal support, resource awareness, setting limits
Moynihan et al. (2011)	USA	56 female college students (mean age = 19 years)	Experimental	Bystander theory	Secondary and tertiary interventions	Behavioural intention	To assess if teaching participants how to be proactive bystanders would help increase their willingness to intervene in instances of IPV.	Results indicated that program participants showed increased bystander efficacy, confidence, responsibility for ending violence and likelihood to help. However, there were no differences between control and program in attitudes expressing denial of the problem over time.
Muralidharan & Kim (2019)	India	98 adults (52% female; mean age = 43.77 years)	Experimental	N/A	Secondary intervention	Behavioural intention	Based on SCT, this study explored whether narrative health messages might prompt bystanders to intervene when they encounter domestic violence.	It was found that narratives had a stronger impact reporting intention than non-narratives and such effects were mediated by feelings of empathy. More importantly, the mediating effects of empathy were significantly greater when bystander efficacy was low rather than high.
Muralidharan & La Ferle (2020)	India	104 adults (51.9% female; mean age = 42.37 years).	Experimental	Psychological distance	Secondary intervention	Behavioural intention	This study explored the persuasive impact of emotional ad appeals—shame (other-focused) and hope (ego-focused)—on varying levels of perceived peer support among participants from India.	The findings showed that hope was more effective than shame in generating favourable attitudes toward the ad and stronger reporting intentions. Furthermore, hope was more effective among those with low perceived peer support, while either emotion worked well for those with high perceived peer support.
Muralidharan, La Ferle & Howard (2020)	India	72 adults (36.4% female; mean age = 41 years)	Experimental	N/A	Secondary intervention	Behavioural intention	This exploratory study tested the ability of public service announcements to inspire behaviour change (*i.e.*, to call a helpline). The current study examined the impact of self-focused emotional appeals, namely guilt (negative) and hope (positive), on varying levels of self-construal (independent *vs.* interdependent).	Guilt and hope were persuasive only on the independent self-construal (not interdependent). Hope (*vs.* guilt) significantly strengthened the intentions to call the advertised helpline for those with low independent self-construal. While, both hope and guilt were found to be equally effective on the high independent self-construal.
Pagliaro et al. (2021)	Italy	110 adults (73% female; mean age 34.07 years, *SD*= 12.97)	Correlational	Attribution theory	Tertiary intervention	Behavioural intention	This article examined indirect consequences for the survivors of IPV in terms of ostracism, reputational threats, and reduced help.	Results show that survivors of IPV (vs. generic violence) received a more negative moral evaluation and considered as more responsible for the violence which was associated with less willingness to approach and defend the survivor or include her in relevant ingroups 1 year later.
Rai (2020)	USA	468 adults (56% female; ages range 18–35 years)	Correlational	Intersectionality theory	Tertiary intervention	Behavioural intention	This study aimed to explore the correlates of recommending a help-seeking resource to a survivor of IPV among the US South Asian community.	Women were more likely to recommend a help-seeking resource than men. However, Individuals who were religious, from a joint family (versus a nuclear family), and those with conservative gender role attitudes had a lesser likelihood of recommending a help seeking resource.
Riley & Yamawaki (2018)	USA	184 college students (59% female; mean age = 20.59 years)	Experimental	N/A	Tertiary intervention	Behavioural intention	This study explored factors that predict an informal supporter’s intentions to help a female IPV survivor in a heterosexual relationship and factors related to victim blaming. Specifically, the effects of Right-Wing Authoritarianism, Benevolent Sexism, and Hostile Sexism, on intentions to help an IPV survivor were investigated.	Participants with higher scores on RWA, BS, HS, those who had the “stay” condition (survivor remained in the violent relationship) and men where all less likely to offer helpful support the victim. Participants with higher scores on RWA, BS, and HS were more likely to respond to survivors in unhelpful ways (*e.g.*, insist that the victim attend couples counselling, advise the survivor she should not make her husband angry).
Storer, McCleary & Hamby (2021)	USA	39 emerging adults (49% female; age range 17–22)	Qualitative	Situational cognitive model of adolescent bystander behaviour	Secondary intervention	Behavioural intention	The purpose of this exploratory study was to investigate emerging adults’ willingness to use bystander behaviours in response to witnessing dating and community violence.	Barriers to intervening were a perception that intervening is dangerous/ snitching, perceived injunctive norms about intervening (*i.e.*, you should “mind your business”), perceived inability to effect change, and struggling “to get it all together” (feel unable to help as they cannot cope with their own stress/struggles). While factors influencing Intervention were the perceived seriousness of the incident, witnessing male-perpetrated forms of dating abuse, relationship to the target or perpetrator of abuse.
Waterman et al. (2021)	USA	899 college students (70.6% female; mean age = 19.5 years, *SD*= 1.2 years)	Correlational	Theory of planned behaviour	Tertiary intervention	Actual behaviour	This paper aimed to examine the association between disclosure recipients anticipated and actual responses to IPV.	Participants who anticipated a higher likelihood of providing negative and positive reactions tended to give those reactions more frequently during a subsequent disclosure. There were also significant positive associations between anticipated and actual victim responsibility, empathy, confusion, and ineffectiveness.
Weitzman, Cowan & Walsh (2020)	USA	1,307 nationally representative adults aged over 15 years	Correlational	Bystander theory	Secondary and tertiary interventions	Actual behaviour	This article investigated the perceived barriers to intervening in IPV.	The study found that fear of physical injury, not wanting to intervene in private matters and fear of misinterpreting the situation were associated with reduced help-giving intention.
Woods et al. (2020)	USA	393 college students (79.7% female; mean age = 19.02 years, *SD*= 2.86)	Correlational	Bystander theory	Secondary and tertiary interventions	Actual behaviour	This study aimed to consider how personal history of recent physical, sexual, and psychological victimisation interacted with likelihood to intervene.	Survivors of psychological aggression were significantly more likely to intervene in low-risk, high-risk, and post-event situations. Survivors of physical and sexual violence were more likely to intervene in low-risk situations.

### Search strategy

The search strategy was developed by the research team (RD, KR, AR) in consultation with a librarian, following a review of other systematic reviews of IPV. Search terms focused on identifying the occurrence of IPV, and also identifying the presence of an informal supporter. Search terms are presented in Table 2. Searches were conducted using the search terms across title and abstract.

**Table 2:** Search terms and items retrieved from each database.

Name of database	Last date accessed	Search terms	Total retrieved
PsychARTICLES	15/12/2021	((“partner violence” OR “partner abuse” OR “domestic violence” OR “domestic abuse” OR “partner assault” OR “domestic assault” OR “battered wife” OR “battered spouse” OR “spouse abuse” OR “spouse assault”) AND (“informal support” OR “family support” OR “social support” OR “family member” OR “social network” OR “neighbour” OR “friend” OR “bystander”))	1,327
Scopus	12/12/2021	((“partner violence” OR “partner abuse” OR “domestic violence” OR “domestic abuse” OR “partner assault” OR “domestic assault” OR “battered wife” OR “battered spouse” OR “spouse abuse” OR “spouse assault”) AND (“informal support” OR “family support” OR “social support” OR “family member” OR “social network” OR “neighbour” OR “friend” OR “bystander”))	698
ProQuest Social Services Abstracts	18/12/2021	((“partner violence” OR “partner abuse” OR “domestic violence” OR “domestic abuse” OR “partner assault” OR “domestic assault” OR “battered wife” OR “battered spouse” OR “spouse abuse” OR “spouse assault”) AND (“informal support” OR “family support” OR “social support” OR “family member” OR “social network” OR “neighbour” OR “friend” OR “bystander”))	223
EbscoHost	07/12/2021	((“partner violence” OR “partner abuse” OR “domestic violence” OR “domestic abuse” OR “partner assault” OR “domestic assault” OR “battered wife” OR “battered spouse” OR “spouse abuse” OR “spouse assault”) AND (“informal support” OR “family support” OR “social support” OR “family member” OR “social network” OR “neighbour” OR “friend” OR “bystander”))	612

### Study selection

To determine if a study met the inclusion criteria, two reviewers (RD and a research assistant) independently evaluated eligibility using a two-step process. First, both reviewers individually screened each study by title and abstract in the Covidence systematic review software (Veritas Health Innovation, 2021). The Covidence systematic review software automatically notified the reviewers when a conflict occurred. In instances where a conflict occurred, a discussion was held between the reviewers to reach a consensus using the systematic review protocol as a guide. On each occasion a consensus was reached without the need for independent evaluation. Should a consensus not have been reached conflicts would have been resolved by the senior researcher (KR). The same review process was utilised in the subsequent full-text screening phase.

### Data extraction

A standardised data collection form was developed by RD, adapted from the Cochrane data collection grid (Higgins et al., 2019). RD extracted all the data from the studies, which is presented in Table 1, and KR and AR reviewed the data.

### Risk of bias

Assessments of the quality and risk of bias of each included study was independently carried out by two reviewers. As the studies included both quantitative (experimental and observational) and qualitative designs, the Mixed Methods Appraisal Tool (MMAT; Hong et al., 2018) was utilised. Each of the included 31 studies was found to have suitable quality and low risk of bias, as per the MMAT scoring system, and therefore were all included in the synthesis. A summary table of the MMAT is provided as a supplementary file.

## Results

### Information about studies selected

**Figure 1:**
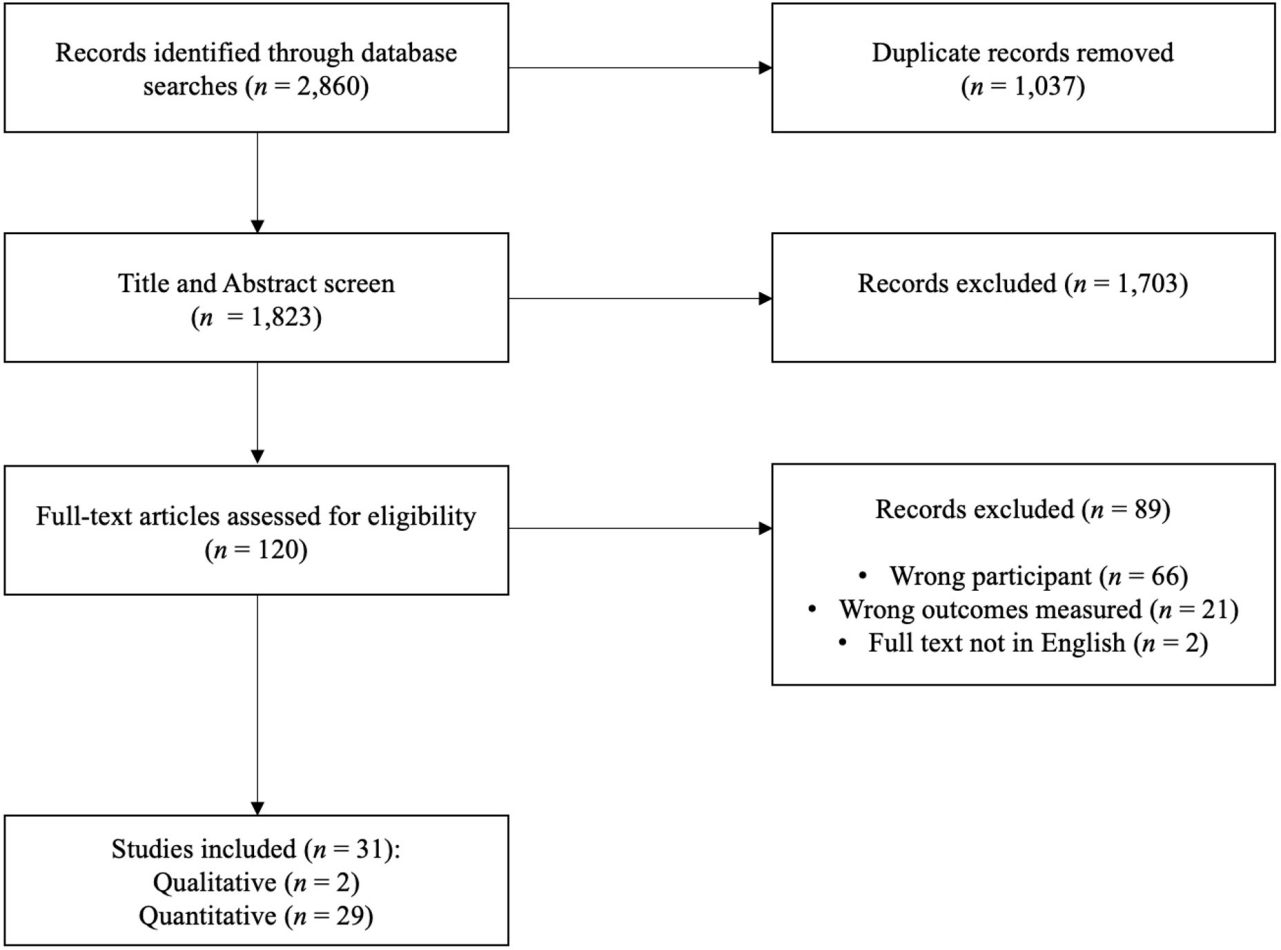
Flow diagram of included and excluded articles.

The initial search retrieved 2,860 articles. Of these articles 1,037 were duplicates and a further 1,793 were assessed as irrelevant based on the screening eligibility criteria. Therefore, 120 articles were screened using the full text resulting in 31 articles being selected for inclusion in the systematic review. An overview of the screening stages is presented in Fig. 1.

Of the 31 studies included in this review, 29 were quantitative and two were qualitative. Of the quantitative studies, 17 were cross-sectional and 12 were experimental. All studies were published between 2005 and 2021. Nineteen studies were conducted in the United States, four in Italy, three in India, three in the United Kingdom, one in Uganda, and one in both the United States and India. The total sample size of all studies was 18,739. Of the total sample, 36.8% of participants were college students and 58.5% were female. Samples ranged between 18 and 6,010 participants. A total of 16 (52%) studies used measures of behavioural intention, 11 (35%) measured occurrences of actual behaviour, and four (13%) measured both behavioural intention and occurrences of actual behaviour. In total 81.5% of participants (*n* = 15,261) reported on behavioural action (*i.e.,* actual helping behaviour as opposed to future intention).

### Key findings from the systematic review

In relation to objective one, the review of identified factors positively or negatively associated with help-giving intention produced three overarching domains. The first domain, normative helping factors, related to an individual’s interpretation of social norms of help-giving in an IPV context. The second domain identified factors associated with individual beliefs about help-giving. The third domain included situation specific factors, such as an individual’s views and beliefs surrounding the survivor, the perpetrator, and the unique pattern of IPV. In relation to the second objective, there were no articles that considered self-care strategies employed by informal supporters. In relation to objective three, nine theories were applied across 22 of the articles. However, none of these theories adequately account for the unique and differing context specific social factors, identified in objective one, that are inherent to IPV, rather they focus on the broader idea of IPV.

### Post-Hoc analysis of secondary and tertiary interventions

Following review of the included papers it was found that a majority of studies, 58% (*n* = 18), had used a single measure which explored both secondary and tertiary interventions together, thus, conflating these types of intervention. This was unexpected given the different barriers faced by informal supporters when providing secondary interventions, which have a greater risk to self, compared to tertiary interventions which occur post-assault when the situation is no longer an ‘emergency’.

### Normative helping factors

The first factor discovered from the results was normative helping beliefs. Normative beliefs, also referred to as subjective norms, are an individual’s evaluation of the beliefs held by other people. Primarily, subjective norms relate to the interpretation of whether people who are important to you would support or condone a target behaviour (Ajzen, 2000). The role of subjective norms on influencing our behavioural intention has been widely studied (for an overview see Chung & Rimal, 2016). It is proposed that social norms are associated with behavioural intention through a desire to fit in with the group identity (Dempsey, McAlaney & Bewick, 2018). This finding is consistent with help-giving behaviour where a desire to conform to group identity benefits the individual through reciprocal altruism; that is, the notion that ‘if I have been helped, then I should help’, and so on (Manatschal & Freitag, 2014). This systematic review identified two social norms that are correlated with help-giving intention regarding IPV survivors (as identified in Baldry & Pagliaro, 2014; Edwards et al., 2014; Storer, McCleary & Hamby, 2021). These norms are categorised as injunctive, which are an individual’s beliefs about what those around them think appropriate behaviours are, and descriptive, which are an individual’s interpretation of the actual behaviours of those around them.

Storer, McCleary & Hamby (2021) found, in their study of low opportunity youth and young adults, that the injunctive norm of “mind you own business” was a substantial barrier to intervening in an incidence of IPV. This perception occurred in the context of high levels of community violence and racially motivated police violence in which intervening could be potentially dangerous. Edwards et al. (2014) measured the injunctive norm of collective efficacy (the perceived level of social cohesion among community members) in 16 rural counties and found that the greater an individual perceived the members of their community as being helpers the more likely they were to offer help.

In their study on descriptive norms, Baldry & Pagliaro (2014) presented a scenario of IPV to participants and then measured their willingness to help. Participants were split into two groups. Participants of group one were advised that most people who were of the same ethnic background as themselves indicated they had helped the survivor (helping condition). The second group were advised that most people of the same ethnic background indicated that although IPV was wrong they would not intervene as relationships are a private matter. Baldry & Pagliaro (2014) found that descriptive norms which suggest helping is morally right are positively correlated with the willingness of an individual to help during an incident of IPV.

The strength of subjective norms is influenced by an individual’s sense of belonging (Allen et al., 2021). An individual’s sense of connection or belonging to the ‘group’ is important in determining the strength of the association between subjective norms and helping intention (Dempsey, McAlaney & Bewick, 2018). In their norms-based study, Baldry & Pagliaro (2014) found that whilst a ‘helping norm’ was associated with intention to help, the level of helping intention was higher when the individual had a stronger sense of belonging to the normative group (*i.e.,* being Italian). Banyard (2008) explored this correlation further and found that a greater perceived sense of connection to the local community was also associated with increased willingness to help and reduced feelings of ineffectiveness. Thus, while the interpretations of others’ thoughts on helping (injunctive norms) and beliefs about others’ actual helping behaviour (descriptive norms) are important, the sense of belonging of the individual appears to have an important association with behavioural intention.

### Individual helping factors

The second factor to be derived from the results of this systematic review was the individual factor, which is related to an individual’s beliefs about help-giving. This factor consists of four distinct sub-factors which are described below.

#### Self-Efficacy

The first sub-factor identified from the results on individual helping intention was self-efficacy. Self-efficacy has been widely studied and has often been found to be a strong predictor of behavioural intention (Luszczynska et al., 2011). A positive association between self-efficacy and altruistic behaviours in general has also been found (Alessandri et al., 2009). Related to IPV, Banyard & Moynihan (2011) found that an individual’s perceived efficacy was positively correlated with bystander intervention behaviours. Banyard (2008) also found that interpersonal control (*i.e.,* the belief that as an individual you can control a situation) and socio-political control (*i.e.,* the belief that active participation in political and social affairs can promote social change broadly) were both associated with more positive bystander outcomes.

Self-efficacy is also an important factor when a disclosure is made to an informal supporter following an act of IPV. In a recent study, an individual’s belief that their response to a disclosure would be positive or negative was correlated with their actual response when later receiving a disclosure (Waterman et al., 2021). Upon becoming aware of IPV, an individual’s confidence in dealing with IPV has been found to partially mediate the relationship between awareness of IPV and the number of positive actions enacted in response (Bovill & White, 2020). Conversely, a perceived inability to effect changes amid feelings of being unable to cope with one’s own stress/struggles was found to reduce the behavioural intention of helping a IPV survivor (Storer, McCleary & Hamby, 2021).

Self-efficacy as a bystander has been identified as an important factor in promoting behavioural intention to help a survivor of IPV, and many IPV awareness programs have targeted increasing bystander self-efficacy. For example, Gainsbury, Fenton & Jones (2020) found that bystander program participation increased self-efficacy of participants, which improved behavioural intent to help. In addition, participants had more favourable views that their peers would be more effective helpers, thereby, increasing the participants’ normative helping perceptions. Bystander programs aim to provide participants with education about IPV and tangible strategies to use to respond to situations of IPV. This increase in confidence in how to respond to IPV situations has been found to increase willingness to intervene (Amar, Sutherland & Kesler, 2012). Self-efficacy has been developed within a bystander program by first practicing the skills learnt for responding to IPV within a safe environment (Moynihan et al., 2011).

#### Acceptability of IPV

Attitudes towards the acceptability of the use of violence in relationships was uncovered in the results of this systematic review as sub-factor associated with IPV help-giving intention. Riley & Yamawaki (2018) found that the beliefs individuals hold about gender roles and norms influenced their helping intention, and that individuals with conservative gender role attitudes had a lesser likelihood of recommending formal support services to the survivor following a disclosure of IPV. Further, Cinquegrana, Baldry & Pagliaro (2018) found that the greater identification of traditional male gender role norms that were held the greater the individual would ascribe responsibility for the IPV to the survivor, thereby, reducing their willingness to offer help. In relation to beliefs of inequality between men and women, Riley & Yamawaki (2018) found that participants with higher scores on measures of both benevolent sexism and hostile sexism were less likely to offer help to a survivor. However, in the event that those with higher scores of benevolent or hostile sexism were to offer support to a survivor they were more likely to respond to survivors in unhelpful ways (*e.g.*, insist that the survivor attend couples counselling, advise the survivor she should not make her husband angry).

Gender norms have been found to be associated with attitudes towards IPV. For example, gender norm beliefs which are less tolerant of violence have been found to be significantly positively associated with enacting informal social control (Frye, 2007; Latta & Goodman, 2011). In their study of low-income community members in Uganda, Abramsky et al. (2018) found that willingness to help survivors was associated with clear attitudes that condemn IPV. Gainsbury, Fenton & Jones (2020) considered the construct of IPV attitudes and explored IPV myths as a means of understanding IPV related attitudes. The identified myths used primarily related to the minimisation of the frequency of violence (*e.g.*, “domestic violence does not affect many people”) and responsibility of the violence (*e.g.*, “making a man jealous is asking for it”). Additionally, individuals who hold strongly endorsed views that IPV is a criminal justice issue were more willing to help survivors of IPV (Beeble et al., 2008). Beeble et al. (2008) described this increased willingness to help as a reflection of the view that IPV is a serious social issue, therefore. requiring intervention. Conversely, Edwards & Dardis (2016) found that more accepting attitudes towards IPV explained the most variance in negative social reactions (*e.g.*, dismissing the survivor).

#### Social responsibility

Next, a felt sense of social responsibility was identified in this systematic review to be a significant factor predicting informal social control (Chaurand & Brauer, 2008). Social responsibility also appears to be present specific to help-giving intention. Banyard & Moynihan (2011) found that individuals who had a greater sense of responsibility for ending IPV exhibited a greater number of helping behaviours. Building a strong social sense of responsibility has also become a key focus of IPV bystander programs. In studies completed by Amar, Sutherland & Kesler (2012) and Moynihan et al. (2011), it was found that an individual’s sense of responsibility to reduce the impact of, and ultimately end, IPV was associated with increased willingness to intervene and help in an IPV context. Conversely, Weitzman, Cowan & Walsh (2020) found that viewing IPV as a private matter was a barrier for informal supporters to take responsibility for ending violence. Additionally, they found that a fear of having misinterpreted the situation (and it not actually being IPV) also reduced the helper’s sense of responsibility.

#### Experiences of violence

An individual’s own experience of violence was identified from the results of the systematic review as being associated with help-giving intention. Chabot et al. (2009) found that participants were more likely to intervene if they had experienced childhood abuse (Chabot et al., 2009). In their quantitative research, Latta & Goodman (2011) found that participants who had experienced child abuse or adult IPV felt their experience helped them to be a better source of support to the survivor. Additionally, Cascardi et al. (2018) found that prior IPV and child victimization were positively associated with proactive behaviours (*i.e.,* building knowledge on IPV and spreading awareness of IPV as a problem). Furthermore, prior IPV victimisation was associated with interventions in risky situations (*i.e.,* confronting problem behaviours and potential perpetrators) and proactively planning to reduce the risk of abuse in social situations. Beeble et al. (2008) found that prior IPV victimisation resulted in informal supporters being more willing to provide instrumental support (*e.g.*, providing accommodation, financial assistance) in addition to emotional, and informational support (*e.g.*, advice and guidance to cope with the situation) than individuals who had not experienced IPV. Beeble et al. (2008) hypothesised that the increased support may be a result of survivors being more knowledgeable about what is most helpful to women experiencing abuse.

Woods et al. (2020) found that the type of violence experienced was associated with responses to different severities of IPV. They found that individuals who had experienced psychological aggression in previous intimate relationships were significantly more likely to intervene in both low and high risk IPV situations as well as post-assault to support the survivor. However, individuals who had experienced physical or sexual violence were more likely to intervene only in low-risk situations.

Finally, knowing someone who has experienced IPV victimisation was found to be associated with greater help-giving intention. Franklin, Brady & Jurek (2017) identified that having known someone who had experienced IPV was associated with greater willingness to help, while Banyard (2008) found that knowing a survivor of sexual violence was associated with greater help-giving. Both researchers postulated that prior knowledge and experience with survivors of IPV might be associated with greater accuracy in identifying IPV in other situations and greater confidence and knowledge regarding how to appropriately respond.

### Situational helping factors

The third and final factor to extracted from the data from this systematic review was situational helping factors, which relate to the individual’s interpretation of the unique elements of the IPV that is occurring. The situational sub-factors include an evaluation of the survivor and the perpetrator. Situational helping factors will vary depending on the context of the IPV occurring.

#### Relationship

The first sub-factor that was discovered from this systematic review concerned relationships. Unique to informal supporters in the context of IPV is that informal supporters will have a pre-existing relationship with the survivor, the perpetrator, or both. Edwards & Dardis (2016) identified that the higher the quality of the relationship to the survivor the more likely an informal supporter was to help them. Storer, McCleary & Hamby (2021) found that in cases where an informal supporter would be required to physically intervene, the relationship with the survivor needed to be quite strong, with participants discussing intervening on behalf of family members and close friends only. Similarly, Weitzman, Cowan & Walsh (2020) highlighted that individuals were less likely to help distant social network members. Latta & Goodman (2011) also found that the emotional closeness of the relationship was important; however, the physical proximity between the informal supporter and the survivor was also important. Conversely, having a less close relationship with the perpetrator was also important (Edwards & Dardis, 2016; Storer, McCleary & Hamby, 2021).

#### Abuse

This systematic review identified that specific contextual details of the abuse are relevant when deciding to intervene. The first element of abuse identified was the frequency. This element comprises consideration of both the current frequency of disclosed IPV incidents and the frequency of lifetime disclosed IPV incidents experienced by the survivor, with both having a positive relationship with help-giving intention and positive social reactions (Edwards & Dardis, 2016).

The second element of abuse identified in the systematic review was the severity. Chabot et al. (2009) found that participants had a greater likelihood of intervention as the severity of abuse increased. In Latta & Goodman’s (2011) research, they found that at the point that a perpetrator had made a threat to kill the survivor or had caused injuries that endangered her life, the seriousness of the situation invoked a helping response from informal supporters. However, in situations where the violence was not life-threatening and had been recurrent for an extended period, informal supporters appeared resigned to it. Storer, McCleary & Hamby (2021) also found that the severity of the violence was an important factor in deciding what action to take when there was also a threat to self. For example, if there was a perceived threat to self and the severity to the survivor was not substantial no action was likely; however, if the severity to the survivor was substantial then the supporter might call the police and report the incident.

#### Responsibility

Attributions of responsibility were identified in this systematic review to be associated with help-giving intention. Edwards & Dardis (2016) found that attributing responsibility for the violence to the survivor was associated with reduced help-giving intention. Furthermore, not only was assigning responsibility associated with reduced help-giving, it was also positively correlated with the likelihood of a negative social reaction following disclosure. Waterman et al. (2021) found that the relationship between attributions of responsibility and anticipated likelihood to help was also present in actual responses when participants were faced with real world incidents of IPV. Attributions of responsibility appear to have lasting impacts on survivors. In their study, Pagliaro et al. (2021) highlighted that survivors’ of IPV were considered to be more responsible for the violence they experienced than people who had been subject to other forms of violence. This attribution of responsibility resulted in less willingness to approach and defend the survivor or include her in relevant ingroups one year later.

There have also been specific factors identified which are associated with increased attribution of responsibility. Riley & Yamawaki (2018) found that a survivor remaining in a violent relationship increased perceptions of responsibility, while Latta & Goodman (2011) found that substance use by the survivor was also associated with increased perceived responsibility. Finally, if the survivor had admitted to infidelity, individuals have been found to be less willing to help (Baldry, Pacilli & Pagliaro, 2015; Cinquegrana, Baldry & Pagliaro, 2018).

#### Empathy

Empathy was also identified in this systematic review as a situational factor and can be defined as the experience of a sympathetic emotional response and concern for someone who is experiencing distress (Dovidio et al., 2006). The experience of empathy has been suggested to drive motivation to lessen the suffering experienced by others, and, subsequently, is considered to motivate helping behaviour in a variety of situations. Both Edwards & Dardis (2016), and Waterman et al. (2021), found that greater feelings of empathy at the time of disclosure of abuse from a survivor were positively associated with providing a positive response and greater help-giving intention. Latta & Goodman (2011) found that greater empathic understanding of the survivor’s mental health was associated with helping intention, and that informal supporters had more empathic responses when the survivor had children.

#### Risk

Another key variable that was uncovered as a situational factor in the present study’s results is the perceived level of risk posed by the perpetrator. Perception of risk level includes consideration of both the risk that intervening might pose to the survivor (*i.e.,* concern for future reprisal) and the risk posed to the informal supporter themselves (Latta & Goodman, 2011). Weitzman, Cowan & Walsh (2020) found that the perceived fear of injury was commonly considered by network members, with 43% of respondents indicating they would be reluctant to help for this reason. Additionally, Storer, McCleary & Hamby (2021) found that not only was risk to self a factor in deciding to intervene in acts of IPV, but also what risks and ongoing consequences might be present from the perpetrator to the informal supporter’s family.

#### Change readiness

The perceived level of change readiness of the survivor to receive support was also identified as a situational factor. Latta & Goodman (2011) found that an informal supporter’s perception of the level of change readiness of a survivor to acknowledge the violence and receive help was associated with help-giving intention. Latta & Goodman (2011) also found that a survivor’s use of substances was an indicator to network members that the survivor was not ready for change or to take action, and this perception was associated with reduced help-giving intention.

#### Emotional response

A range of emotional responses experienced when IPV is identified by the informal supporter discovered in this systematic review as a sub-factor of situational helping intention. For example, the immediate emotional distress experienced by the informal supporter was found to be associated with both negative and positive social reactions to disclosures of IPV. Specifically, informal supporters who experienced emotions such as anger and frustration were associated with exhibiting negative social reactions. While emotions of informal supporters such as shock and fear were able to motivate positive social responses if they elicited feelings of empathy (Edwards & Dardis, 2016).

Feelings of hope for the survivor and the situation have been associated with increased willingness to help survivors. Specifically, feelings of hopefulness have been positively correlated with informal supporters contacting a violence helpline to seek guidance on how to respond (Muralidharan & La Ferle, 2020). Feelings of hope were also found to moderate the relationship between level of perceived peer support and help-giving intention (Muralidharan & La Ferle, 2020). Additionally, advertisements that targeted feelings of guilt and shame were more impactful on reporting intention than control groups (La Ferle, Muralidharan & Kim, 2019). Lastly, feeling overwhelmed by the disclosure, in addition to feeling overwhelmed by their own current stressors, was associated with reduced help-giving intention (Storer, McCleary & Hamby, 2021).

Overall, a number of variables were identified as being associated with increases or decreases in an individual’s willingness to support an IPV survivor. The identified variables fall into the three discrete, yet related, domains of normative, individual, and situational factors.

### Self-care of informal supporters

The second objective of this systematic review was to consider how informal supporters utilise self-care strategies and the effectiveness of these strategies. As IPV is often an ongoing chronic pattern of behaviour, informal supporters are regularly subjected to the negative consequences of IPV over long periods of time. Gregory et al. (2017) found in their systematic review of the impacts of help-giving on informal supporters that it was common for informal supporters to experience a range of negative physical and psychological effects. However, this systematic review did not identify any articles that considered how informal supporters manage their self-care in the face of the difficulties experienced when supporting an IPV survivor.

### Current theoretical conceptualisations

From the 31 articles selected for review, 22 had a theoretical basis for the hypotheses presented and nine articles presented no clear theoretical basis. Of these 22 theory-based articles, nine unique theories were tested. The most common theory presented was Bystander theory, which was applied in 12 articles. Bystander theory was first proposed by Darley & Latané (1968), in response to the (incorrectly) reported lack of bystander action taken by neighbours who could hear a woman being murdered (Kassin, 2017). The theory proposes five stages (*i.e.,* notice the event, interpret as an emergency, assume responsibility, know how to help, and decide to implement help) that must be met for help to be given. Bystander theory has been widely supported in social psychology (Fisher et al., 2020); however, its utility in the context of IPV is limited. For example, bystander theory has largely been used to address responses to acts of violence occurring in public in which neither the perpetrator nor the victim is known to the individual. This is not the case for social network members of survivors who have a relationship with the survivor and often with the perpetrator as well. Bystander theory also does not account for the interpretation of responsibility, as informal supporters in an IPV context will make a determination of responsibility (*e.g.*, the survivor’s actions, such as infidelity, might be attributed as being responsible for causing the violence).

The second most frequent theory was attribution theory, which was used in three articles. Attribution theory in the context of IPV was first explored by West & Wandrei (2002). Whilst primarily focused on attributions of responsibility, Edwards & Dardis (2016) created a model that included situation-specific, individual, relational, attributional, and emotional factors. However, while this model considered the unique context of the violence occurring within an intimate relationship, it did not account for the multiple individual factors that are associated with help-giving intention, such as individual efficacy, ability, and assuming responsibility to stop the violence. An overview of the reviewed theories is included in Table 3.

**Table 3:** Overview of theories utilised in articles presented in this systematic review.

Theory	Original author	Use in IPV context
Attribution theory	Heider (1958)	Attribution theory aims to explain how the social perceiver uses information to arrive at causal explanations for events. In terms of IPV, attributions of responsibility are used to determine which party is responsible for the violence and, therefore, if a survivor is deserving of helpful intervention (Cinquegrana, Baldry & Pagliaro, 2018; Edwards & Dardis, 2016; Pagliaro et al., 2021).
Bystander theory	Darley & Latané (1968)	Bystander theory applies a five-step situational model of bystander behaviour for the decision-making process of individuals to act as a bystander. The steps include noticing the event, interpreting it as an emergency, taking responsibility for acting, deciding how to act, and, finally, choosing to act (Abramsky et al., 2018; Amar, Sutherland & Kesler, 2012; Amar, Sutherland & Laughon, 2014; Banyard, 2008; Banyard & Moynihan, 2011: Bovill & White, 2020; Cascardi et al., 2018; Chabot et al., 2009; Fenton & Mott, 2018; Gainsbury, Fenton & Jones, 2020; Moynihan et al., 2011; Weitzman, Cowan & Walsh, 2020; Woods et al., 2020).
Intersectionality theory	Crenshaw (1991)	Intersectionality theory acknowledges power, status, and identity differences among men and women, and examines how these differences inform their preferences for help-seeking resources and indirect experiences with IPV (Rai, 2020).
Moral disengagement theory	Bandura (1999)	Moral disengagement theory proposes that individuals can cognitively separate moral standards from a particular behaviour in order to avoid applying the moral standard to a particular situation. In terms of IPV help-giving, some individuals cognitively and socially withdraw thinking that it is not up to them to do something (Baldry, Pacilli & Pagliaro, 2015).
Psychological distance	Perloff & Fetzer (1986)	Psychological distance theory identifies a difference between directly experiencing events present in one’s reality, versus those that are beyond the reach of direct experience. In other words, using the ‘self’ as a reference point, it is the distance from the self to an event. Psychological Distance theory outlines the following four dimensions: temporal distance, spatial distance, social distance and hypotheticality. In terms of IPV help-giving, social distance is used to understand action or inaction by the degree of similarity between the self and the comparison member or group (Muralidharan & La Ferle, 2020).
Situational cognitive model of adolescent bystander behaviour	Casey, Lindhorst & Storer (2017)	The situational cognitive model of adolescent bystander behaviour identifies situational, setting-level, interpersonal, and individual factors that influence adolescents’ bystander behaviour. This theory integrates elements from the theory of planned behaviour and bystander theory’s model of bystander behaviour (Storer, McCleary & Hamby, 2021).
Social disorganisation theory	Shaw & McKay (1969)	Social disorganization theory proposes that the level of community wide collective efficacy influences behavioural intention (Edwards et al., 2014).
Social identity theory	Tajfel & Turner (1979)	Social identity theory states that people derive an important part of their identity from the groups to which they belong. Therefore, the more individuals identify with their own group, the more they are inclined to adhere to the group’s norms (Baldry & Pagliaro, 2014).
Theory of planned behaviour	Ajzen (1991)	Theory of planned behaviour proposes that behavioural intention is determined by one’s attitudes towards a behaviour, subjective norms surrounding a behaviour, and perceived behavioural control (Waterman et al., 2021).

## Discussion

This systematic review assessed the evidence of IPV related help-giving behaviour and intentions. The present study also explored the theoretical models that have been utilised to understand this behaviour and the adequacy of these possible explanatory models within the IPV help-giving context. To ensure that all relevant studies were considered both quantitative and qualitative articles were included. Following review, by two independent reviewers, thirty-one articles were included in the systematic review. This systematic review of recent research has identified a range of IPV help-giving factors, including factors that are associated with general help-giving intention, as well as factors specific to the unique context of IPV. While a range of theoretical models were applied throughout the studies, none of these models adequately explain the full scope of behavioural intention to help a survivor of IPV. To address this gap, based on the results of the systematic review, the present authors propose an integrated theoretical model of the factors hypothesised to promote positive behavioural intentions to support IPV survivors.

### Proposed model

**Figure 2:**
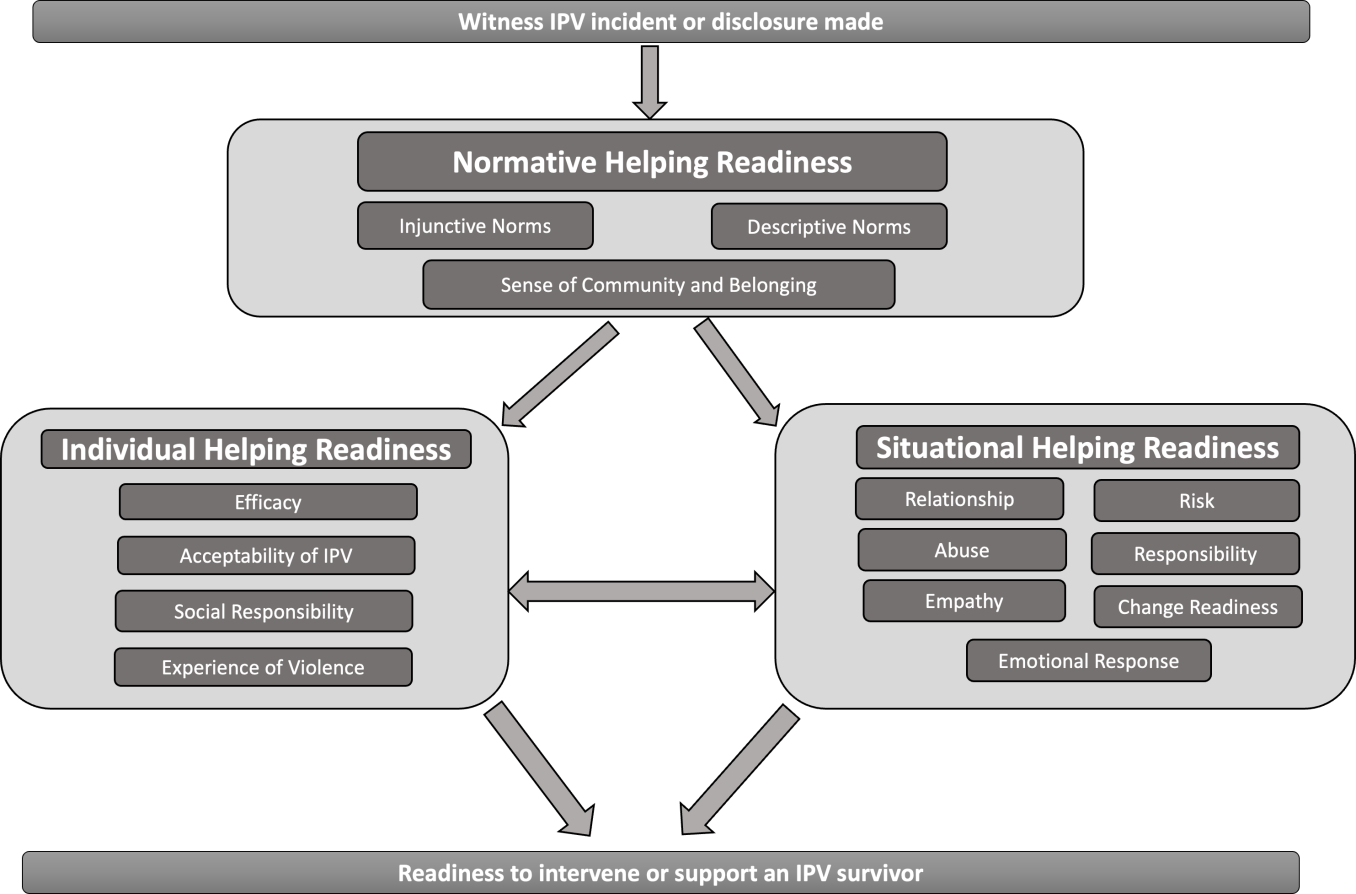
The proposed intimate partner violence model of informal supporter readiness (IPV-MISR).

Following our synthesis of the literature, we propose the Intimate Partner Violence Model of Informal Supporter Readiness (IPV-MISR). This model hypothesises that behavioural intention to support a survivor of IPV is driven by normative helping intentions, individual helping intentions, and situational helping intentions. Each of these factors consists of a series of unique variables that have been found to be associated with help-giving readiness. Figure 2 presents a diagrammatic overview of the model and is followed by a description of these factors. This model will add support to the current understanding of help-giving intention to survivors of IPV; however, it is noted that decisions around support for IPV survivors should be centered on the individual survivor’s views and needs.

### Normative helping readiness

(1) *Injunctive Norms* guide individual behaviour by presenting perceptions of which behaviours are typically approved or disapproved. Therefore, injunctive norms assist in determining what is acceptable and unacceptable social behaviour. In the context of IPV, injunctive norms aid an individual to conceptualise whether intervening would be a positive step towards helping a survivor or would be a social misstep that would be interfering in a private matter.
(2) *Descriptive Norms* are real world, observed behaviours of significant others in the target situation. How significant others respond to situations of IPV adds evidence to the development of our view of helping survivors of IPV as either beneficial or inappropriate.
(3) *Sense of Community and Belonging:* the strength of injunctive and descriptive help-giving norms is correlated with the level of motivation and an individual has to comply with those norms. Therefore, an individual who has a stronger sense of belonging and connection to their community will have a greater tendency to conform to injunctive and descriptive norms.

### Individual helping readiness

(1) *Self-efficacy* is required to ensure that the informal supporter has the confidence that they can effectively help, whilst also having the capacity, skills, and knowledge to do so.
(2) *Acceptability of IPV* can influence our view of the value of help-giving. Beliefs of traditional gender norms can foster beliefs that IPV is a private matter and, therefore, not to be interfered with. Both benevolent and hostile sexist attitudes contribute to the belief that survivors are to blame for the violence (even if partially) and, therefore, are not worthy of receiving help.
(3) *Social Responsibility*: An individual’s belief that they have a responsibility to intervene in an IPV situation is associated with behavioural intention. This belief can be formed from a sense of justice, and this responsibility coupled with other factors (*e.g.*, the relationship to the survivor) can be used to predict positive interventions.
(4) *Experience of violence* can influence the level of emotional preparedness one has to respond to IPV. Additionally, prior experience can aid in developing practical skills on how to provide support and what support might be most appropriate.

### Situational helping readiness

(1) *Relationship*: Individuals are more willing to expend personal resources to aid those with whom they have a stronger relationship. A strong relationship to the survivor is particularly important in the context of chronic IPV, where an informal supporter may be required to provide ongoing (even if sporadic) support to a survivor for an extended period.
(2) *Abuse:* An evaluation of the severity, frequency, and chronicity of the abuse helps to determine the overall impact of the abuse on the survivor and, therefore, the response needed. The greater the impact, the greater the need for intervention.
(3) *Responsibility:* Greater help-giving intention occurs when an individual has a clear position that the perpetrator is responsible for the violence. If the informal supporter attributes responsibility to the survivor (*e.g.*, apporting blame due to infidelity) they become less worthy of help.
(4) *Empathy:* The sense of sympathetic wrongdoing to a survivor can motivate an informal supporter to act. An emotional connection to the survivor gives the informal supporter a sense of moral obligation to provide help.
(5) *Risk:* Greater levels of perceived risk (to the survivor, the supporter, or others), will reduce the behavioural intention to help as the risk outcome might be more significant than the benefits of providing support.
(6) *Change Readiness:* Informal supporters must decide if the cost of providing intervention will be well received and utilised by a survivor (*i.e.,* the survivor is ready for change), therefore, making the informal supporter’s efforts useful.
(7) *Emotional Response*: an individual’s emotional response can be linked to the perception that that their intervention will be useful. Emotions of hope are linked to long term goal success, which is important given the generally on-going nature of IPV before a survivor is safely able to remove themselves from a perpetrator (if this is ever possible).

### Implications

The findings of this systematic review, and the subsequent development of the IPV-MISR, are a significant contribution to bridging the gap in current conceptualisations of help-giving in the context of IPV. With a focus on situational helping readiness, the IPV-MISR will contribute to extending research in this area by allowing for greater sensitivity to the nuanced social constructs inherent in IPV, which influence help-giving.

Specifically, these findings could be utilised in research and practice to refine bystander intervention programs. Currently, bystander programs are based on a bystander theory conceptualisation of help-giving and could benefit from greater consideration of the nuanced factors associated with IPV, as identified in this review. For instance, by incorporating additional exercises, such as self-reflection on the strength of relationships, bystander programs would be able to promote positive change across a greater number of helper readiness factors.

Being guided by the domains of readiness described above, consideration of how to improve interventions to promote tertiary informal supporter readiness could also consider how to promote ongoing positive bystander behaviours in the face of chronic IPV post-disclosure of abuse (which is the more common occurrence), as opposed to bystander behaviour in the context of witnessing an ‘urgent’ situation. Building the continuing capacity for informal supporters to safely engage with IPV survivors is important for the ongoing support needs of a survivor.

It is anticipated that the findings of this systematic review, and subsequent development of the proposed model of helping, will have real world implications in strengthening the social support systems of survivors. Through application of a network orientated approach (Ogbe et al., 2020), the current model can be utilised by both informal supporters and by IPV safety advocates (*e.g.*, shelter workers, IPV outreach workers), to work collaboratively with survivors and their social network members to assess the readiness and willingness of informal supporters to provide help. This reflective process can be utilised to identify areas in which network members might require support themselves to increase their readiness to help survivors.

Finally, one of the purposes of this systematic review was to identify self-care strategies employed by informal supporters. However, this systematic review did not find any articles that evaluated informal supporter self-care strategies. This is noteworthy given the impact that IPV help-giving has on informal supporters (Gregory et al., 2017). In a systematic review on the topic of impacts of IPV help-giving, Gregory et al. (2017) found that help-giving is associated with physical and psychological health impacts, as well as having direct negative impacts from the perpetrator. However, to the best of our knowledge, there is no published study that considers how informal supporters can best maintain their physical and psychological safety.

### Limitations of the review

The current review was limited to studies published in English, of which over half originated in the United States. While there were some studies that had been translated to English for publication, this might have influenced some of the conclusions drawn and limit generalisability to linguistically and culturally diverse groups. Therefore, it is possible that additional culturally specific factors would need to be considered if applying the IPV-MISR to cultural groups not considered in this review. Further, other key demographics such as education level, socio-economic status, and age were inconsistently reported. Therefore, it is unknown what role these demographics play on help-giving intention.

Additionally, the majority of articles in this review, 52% (*n* = 16) explored behavioural intention, as opposed to considering the actual behaviours of informal supporters. While there is evidence that suggests that behavioural intention is a predictor of actual behaviour (Webb & Sheeran, 2006), this has not been examined widely in the IPV informal support literature, with only four articles (13%) in this systematic review considering the relationship between help-giving intention and actual help-giving behaviour. As such, it is recommended that future research use the IPV-MISR in contexts of actual behaviours completed in order to provide validity to the utility of the constructs.

In a similar vein, the majority of articles in this systematic review (*n* = 18, 58%) used measures which included both secondary and tertiary interventions simultaneously. Given the different barriers and risks associated with these types of interventions, it is suggested that future research aims to understand the relationship between the constructs of the Informal Supporter Readiness Model and informal supporter intervention type.

## Conclusions

To our knowledge, this was the first systematic review to consider the factors which are associated with secondary and tertiary help-giving behaviours specific to the context of IPV against women. The 31 included studies identified a range of normative, individual, and situational factors associated with IPV help-giving. The findings have been incorporated into the proposed IPV-MISR. The IPV-MISR is the first model of IPV help-giving intention to account for each of the three identified factors. Additionally, the IPV-MISR advances our current understanding of help-giving intention as it is the first to describe how the complexities of relationships between the informal supporter, the survivor and the perpetrator are associated with help-giving behaviour. The findings of this systematic review and the creation of the IPV-MISR will facilitate the development of a psychometric measure of informal supporter readiness to help IPV survivors, which would have real world applications in supporting survivors to develop strong safety plans with their social networks and reduce the risk of IPV.

##  Supplemental Information

10.7717/peerj.15160/supp-1 Supplemental Information 1 Rationale for systematic review

10.7717/peerj.15160/supp-2 Supplemental Information 2 Risk of Bias Summary Table

10.7717/peerj.15160/supp-3 Supplemental Information 3 Prisma Checklist

## Additional Information and Declarations

### Data Availability

The following information was supplied regarding data availability: This article is a systematic review.
