# Supplementary material for: A systematic review of informal supporters of intimate partner violence survivors: the intimate partner violence model of informal supporter readiness
Source: PeerJ. 2023 May 9;11:e15160. doi: 10.7717/peerj.15160 (PMC10178208; doi:10.7717/peerj.15160)
Supplement: Supplemental Information 1 [file peerj-11-15160-s001.docx]

**A Systematic Review of Informal Supporters of Intimate Partner Violence Survivors: The Intimate Partner Violence Model of Informal Supporter Readiness**

**Supplemental information**

1. The rationale for conducting the systematic review;

Intimate partner violence (IPV) is a serious public health issue that consists of physical, sexual, and psychological violence perpetrated by a current or former partner. Informal supporters (e.g., family and friends) of survivors are more often witness to IPV or are the first people a survivor will disclose abuse to and are more able to provide consistent ongoing support than professional services. Therefore, greater understanding of informal supporters is warranted to aid in reducing the risks experienced by survivors. However, the movement towards a network orientated approach towards survivor safety planning remains a relatively new topic of consideration. Therefore, this systematic review aims to explore the existing body of literature which considers the perspective of informal supporters in order to get an understanding of the factors which motivate or demotivate informal supporters from providing support to survivors or IPV.

1. The contribution that it makes to knowledge in light of previously published related reports, including other meta-analyses and systematic reviews

This systematic review proposes a first of its kind integrated model of help-giving behaviour in the context of intimate partner violence. This model provides a framework for conceptualising the readiness of an informal supporter to provide adequate support to IPV survivors and takes into account the unique nature of situation specific factors. The model extends existing theoretical standpoints and has utility in both practice and research.
