# Supplementary material for: A systematic review of informal supporters of intimate partner violence survivors: the intimate partner violence model of informal supporter readiness
Source: PeerJ. 2023 May 9;11:e15160. doi: 10.7717/peerj.15160 (PMC10178208; doi:10.7717/peerj.15160)
Supplement: Supplemental Information 2 [file peerj-11-15160-s002.docx]

**Mixed Methods Appraisal Tool (MMAT), Version 2018 (Hong et al., 2018)**

| **Qualitative Studies** | **S1** | **S2** | **1.1** | **1.2** | **1.3** | **1.4** | **1.5** |
| --- | --- | --- | --- | --- | --- | --- | --- |
| Latta & Goodman, 2011 | ✓ | ✓ | ✓ | ✓ | ✓ | ✓ | ✓ |
| Storer et al., 2021 | ✓ | ✓ | ✓ | ✓ | ✓ | ✓ | ✓ |

| **Quantitative – Randomised Control Trials** | **S1** | **S2** | **2.1** | **2.2** | **2.3** | **2.4** | **2.5** |
| --- | --- | --- | --- | --- | --- | --- | --- |
| Abramsky et al., 2018 | ✓ | ✓ | ✓ | ✓ | ✓ | ✓ | ✓ |
| Baldry and Pagliaro, 2014 | ✓ | ✓ |  | ✓ | ✓ | ✓ | ✓ |
| Cinquegrana et al., 2017 | ✓ | ✓ | ✓ | ✓ | ✓ |  | ✓ |
| Moynihan et al., 2011 | ✓ | ✓ |  | ✓ | ✓ |  | ✓ |
| Muralidharan & Kim, 2019 | ✓ | ✓ |  | ✓ | ✓ |  | ✓ |
| Muralidharan & La Ferle, 2020 | ✓ | ✓ | ✓ | ✓ | ✓ |  | ✓ |
| Muralidharan et al., 2020 | ✓ | ✓ |  | ✓ | ✓ |  | ✓ |

| **Quantitative – Non-Randomised** | **S1** | **S2** | **3.1** | **3.2** | **3.3** | **3.4** | **3.5** |
| --- | --- | --- | --- | --- | --- | --- | --- |
| Fenton & Mott, 2019 | ✓ | ✓ | ✓ | ✓ |  |  | ✓ |
| Gainsbury et al., 2020 | ✓ | ✓ | ✓ | ✓ |  |  | ✓ |

| **Quantitative – Descriptive** | **S1** | **S2** | **4.1** | **4.2** | **4.3** | **4.4** | **4.5** |
| --- | --- | --- | --- | --- | --- | --- | --- |
| Amar et al., 2012 | ✓ | ✓ | ✓ | ✓ |  | ✓ | ✓ |
| Amar et al., 2014 | ✓ | ✓ |  | ✓ | ✓ |  | ✓ |
| Baldry et al., 2015 | ✓ | ✓ |  | ✓ |  | ✓ | ✓ |
| Barnyard and Moynihan, 2011 | ✓ | ✓ |  | ✓ | ✓ | ✓ | ✓ |
| Beeble et al., 2008 | ✓ | ✓ | ✓ | ✓ | ✓ | ✓ | ✓ |
| Bovill and White, 2020 | ✓ | ✓ | ✓ | ✓ | ✓ |  | ✓ |
| Chabot et al., 2009 | ✓ | ✓ | ✓ |  | ✓ | ✓ | ✓ |
| Edwards & Dardis, 2020 | ✓ | ✓ | ✓ |  | ✓ | ✓ | ✓ |
| Edwards et al., 2014 | ✓ | ✓ | ✓ | ✓ | ✓ | ✓ | ✓ |
| Franklin et al., 2017 | ✓ | ✓ | ✓ |  | ✓ |  | ✓ |
| Frye, 2007 | ✓ | ✓ | ✓ | ✓ | ✓ | ✓ | ✓ |
| La Ferle et al., 2019 | ✓ | ✓ | ✓ | ✓ | ✓ |  | ✓ |
| Pagliaro et al., 2021 | ✓ | ✓ |  | ✓ | ✓ | ✓ | ✓ |
| Rai, 2020 | ✓ | ✓ | ✓ | ✓ | ✓ | ✓ | ✓ |
| Riley & Yamawaki, 2018 | ✓ | ✓ |  | ✓ | ✓ | ✓ | ✓ |
| Weitzman et al., 2020 | ✓ | ✓ | ✓ | ✓ | ✓ |  | ✓ |
| Woods et al., 2020 | ✓ | ✓ |  | ✓ | ✓ | ✓ | ✓ |
| Cascardi et al., 2021 | ✓ | ✓ |  | ✓ | ✓ | ✓ | ✓ |
| Banyard, 2008 | ✓ | ✓ |  | ✓ | ✓ | ✓ | ✓ |
| Waterman et al., 2021 | ✓ | ✓ |  | ✓ | ✓ | ✓ | ✓ |
